# Regulation of Petal Coloration by the Auxin Amide Hydrolase Gene *RhILL1* in Rose (*Rosa hybrida*)

**DOI:** 10.3390/genes16060691

**Published:** 2025-06-06

**Authors:** Dan Wang, Yiping Zhang, Daliang Li, Xujun Ma, Xiao Yang, Hongying Jian, Huichun Wang, Lihua Wang, Hao Zhang, Qigang Wang, Xianqin Qiu

**Affiliations:** 1 Flower Research Institute, Yunnan Academy of Agricultural Sciences, Yunnan Flower Breeding Key Lab, Yunnan Flower Research and Development Center, Kunming 650205, Chinablackfarinj@126.com (Y.Z.); maxjuner@163.com (X.M.); ynwildflower@aliyun.com (H.J.); wanghc1996116@163.com (H.W.); wanglihua2525@outlook.com (L.W.); zhanghao7898@sina.com (H.Z.); 2 Institute of Plant Resources, Yunnan University, Kunming 650091, China

**Keywords:** *Rosa hybrida*, petal color, indole-3-acetic acid (IAA), IAA-leucine resistant1-like hydrolase gene (*RhILL1*), VIGS

## Abstract

Objective: This study aimed to elucidate the regulatory mechanism of an auxin amide hydrolase gene (IAA-Leucine Resistant1-like Hydrolase, *RhILL1*) in the petal pigmentation of rose (*Rosa hybrida*), providing theoretical insight into the hormonal regulation of flower coloration at the molecular level. Methods: Using petals at Stage 3 (S3) of the cut rose cultivar ‘Pink Floyd’ as experimental material, we cloned the rose auxin amide hydrolase gene *RhILL1* and validated its function via virus-induced gene silencing (VIGS). The expression levels of anthocyanin biosynthetic genes, anthocyanin content, and auxin (IAA) levels were analyzed to assess the role of *RhILL1* in petal pigmentation. Results: The full-length open reading frame (ORF) of *RhILL1* was cloned, spanning 1326 bp and encoding a 441-amino-acid protein harboring two conserved domains, Peptidase_M20 and M20_dimer, characteristic of the ILL1 protein family. Functional characterization was performed using VIGS. Quantitative real-time PCR (qRT-PCR) revealed that *RhILL1* expression progressively increased from the Green (G) stage to S3, correlating with intensified petal coloration. Silencing *RhILL1* resulted in visibly lighter petals, the reduced expression of anthocyanin biosynthetic genes, and a significant decrease in endogenous indole-3-acetic acid (IAA) levels compared with controls. Moreover, exogenous application of 10 μM naphthaleneacetic acid (NAA) to petals significantly preserved petal pigmentation. Conclusion: These findings suggest that *RhILL1* contributes to the development and maintenance of petal coloration in rose, likely by modulating IAA levels, thereby influencing the expression of anthocyanin biosynthesis-related genes.

## 1. Introduction

Rose (*Rosa hybrida*) is one of the most widely cultivated ornamental plants worldwide, with its diverse and vibrant petal colors significantly enhancing its aesthetic appeal and market value. The formation of flower color primarily depends on the biosynthesis of pigments such as anthocyanins [1,2]. In recent years, extensive studies have revealed that a variety of transcription factors and metabolic enzymes are involved in regulating anthocyanin biosynthesis [3,4]. With increasing understanding of hormonal regulation in plants, auxin has emerged as a key phytohormone not only in plant growth and development but also in controlling anthocyanin accumulation and pigmentation via complex signaling networks [4,5,6].

The core components of anthocyanin biosynthesis are structural genes, which include early biosynthetic genes (EBGs), such as *chalcone synthase* (*CHS*), *chalcone isomerase* (*CHI*), *flavanone 3-hydroxylase* (*F3H*), and *flavonoid 3′-hydroxylase* (*F3′H*), as well as late biosynthetic genes (LBGs), including *dihydroflavonol 4-reductase* (*DFR*), *anthocyanidin synthase* (*ANS*), and *UDP-glucose:flavonoid 3-O-glucosyltransferase* (*UFGT*) [7,8,9]. These structural genes are transcriptionally regulated by MYB, bHLH, and WD40 proteins, which assemble into the MBW (MYB–bHLH–WD40) complex to promote anthocyanin biosynthesis [10]. Notably, the activities of these transcription factors can be modulated by phytohormones, including auxin [4,11,12].

Auxin plays a central role in numerous plant developmental processes, including cell elongation [13], root development [14], and fruit ripening [15], and is synthesized via both tryptophan-dependent and independent pathways [16]. Auxin signaling is mainly mediated through the TIR1/AFB–Aux/IAA–ARF pathway. In this cascade, Aux/IAA proteins inhibit the activity of auxin response factors (ARFs), thereby repressing downstream gene expression [17]. Upon auxin accumulation, Aux/IAA proteins are targeted for degradation via the ubiquitin–proteasome pathway, releasing ARFs to activate auxin-responsive genes [18].

Although auxin is widely recognized for its role in plant development, its function in regulating floral pigmentation remains incompletely understood. Emerging evidence suggests that the effect of auxin on anthocyanin biosynthesis is species-specific. In various fruit crops, such as raspberry [19], apple [6,20], and grapevine [4,21], auxin has been shown to modulate anthocyanin accumulation. In peach, auxin treatment upregulates several anthocyanin-related genes, including *PpCHS*, *PpDFR*, *PpF3H*, and *PpUFGT* [22]. These findings highlight the complexity of auxin action, which is further influenced by cross-talk with other hormones.

Auxin amide hydrolases, such as IAA-Leucine Resistant1-like Hydrolase (ILL1), are key enzymes in auxin metabolism. They hydrolyze auxin–amino acid conjugates (e.g., IAA-Asp and IAA-Glu) to release free IAA, thereby maintaining cellular auxin homeostasis [23]. Within optimal ranges, auxin can activate ARF transcription factors and synergize with anthocyanin-related regulators or directly influence the expression of transcription factors associated with anthocyanin biosynthesis, subsequently modulating the expression of structural genes [4,6,24]. For instance, in the cherry cultivar ‘Sweet Georgia’, NAA treatment significantly upregulates the expression of *PpMYB10.1-1*, *PpbHLH3*, and *PpPAL*, all associated with anthocyanin biosynthesis [25]. In grapevine, the auxin response factor VvARF3 represses VvFHY3-mediated anthocyanin biosynthesis by forming a transcriptional inhibitory complex [4]. In apple, MdARF13 represses anthocyanin accumulation by interacting with MdMYB10 and inhibiting *MdDFR* promoter activity. High concentrations of auxin promote the degradation of MdIAA121 via the 26S proteasome pathway, thereby enhancing the repressive function of MdARF13 on anthocyanin biosynthesis [6]. This is consistent with the findings of Ji et al. [20], who reported that anthocyanin accumulation was significantly reduced under high levels of 2,4-D or NAA treatment in callus cultures of red-fleshed apple. Conversely, in *Arabidopsis thaliana*, exogenous IAA treatment increases the expression of structural genes (*CHS*, *CHI*, and *F3′H*) and regulatory genes (*TTG1*, *PAP1*, and *MYB12*) involved in anthocyanin biosynthesis, suggesting that auxin can promote anthocyanin synthesis within a defined concentration range [24].

However, whether *RhILL1* regulates rose petal coloration by modulating auxin levels remains unknown. This study aims to elucidate the molecular mechanism by which *RhILL1* influences flower coloration in rose through its impact on auxin homeostasis. Our findings will contribute to a better understanding of hormone-mediated pigment regulation and provide valuable genetic resources for molecular breeding of ornamental plants, such as rose.

## 2. Materials and Methods

### 2.1. Plant Materials and Treatments

The plant material used in this study was the cut rose cultivar ‘Pink Floyd’ (two-year-old plants of *R. hybrida*), cultivated at the Baofeng experimental station of the Flower Research Institute, Yunnan Academy of Agricultural Sciences (Jinning, Yunnan Province, China). Uniform, healthy flower stems with consistent thickness and growth vigor were selected for subsequent virus-induced gene silencing (VIGS) experiments. In the laboratory, the stems were trimmed to a length of approximately 25 cm, retaining one to two compound leaves, and equilibrated in deionized water for 2–6 h prior to use.

For petal disc assays, the outermost petals at Stage 3 (S3) were collected from fully open ‘Pink Floyd’ flowers. Circular discs (1 cm in diameter) were excised from the central region of each petal. The discs were pooled, mixed evenly, and divided equally into two treatment groups for infiltration. For exogenous auxin treatment, the petal discs were placed on 1% agar plates supplemented with either 10 μM or 50 μM 1-naphthaleneacetic acid (NAA). The NAA-containing agar medium was prepared by dissolving NAA in a small volume of ethanol, diluting it with deionized water to the desired concentration, and adding it to molten 1% agar (~50 °C) before pouring it into Petri dishes. The discs were incubated in 1% agar plates under dark conditions at 8 °C for 3 days and then transferred to a growth chamber maintained at 22 ± 1 °C, with a 16 h light/8 h dark photoperiod and a light intensity of 8000 lux [26]. Petal phenotype was documented upon completion of color changes in all discs, and petal color variation was quantified according to the method described by Jing et al. [27].

The samples were wrapped in aluminum foil, snap-frozen in liquid nitrogen, and either immediately used for RNA extraction or stored at −80 °C for later use.

### 2.2. RNA Extraction and Quantitative Real-Time PCR (qRT-PCR)

Samples were collected from various tissues of ‘Pink Floyd’, including roots, stems, leaves, petals, stamens, pistils, sepals, and buds, as well as from petals at distinct developmental stages: Green (G), Pink (P), and Stages 1 through 5 (S1–S5) [27]. Additionally, petals from TRV (Tobacco rattle virus) and TRV-*RhILL1* treatments were sampled. Total RNA was extracted using an EASY spin Plant RNA Kit (centrifugal column type; Wuhan Joinnord Biotechnology Co., Ltd., Wuhan, China), optimized for complex plant tissues with high polysaccharide and polyphenol contents.

The quality of extracted RNA was assessed by 1.5% agarose gel electrophoresis. RNA samples that met the quality criteria were subjected to reverse transcription. Gene-specific primers for qRT-PCR were designed (Supplement Table S1), and cDNA synthesized from total RNA was used as a template. Gene expression levels were quantified using SYBR-based qRT-PCR assays, following the manufacturer’s protocol. The RhUBI2 gene was used as an internal reference, and relative expression levels were calculated using the 2^−ΔΔCT^ method.

### 2.3. Gene Cloning

Based on the reference genome sequence of Rosa chinensis ‘Old Blush’ (https://lipm-browsers.toulouse.inra.fr/pub/RchiOBHm-V2/) (accessed on 31 July 2023), gene-specific forward and reverse primers were designed using SnapGene software (from Insightful Science; available at snapgene.com) (accessed on 11 October 2023) (Supplement Table S1). Total RNA was extracted from rose petals using a Plant RNA Kit (Omega Bio-Tek, Norcross, GA, USA), and 1 μg of RNA was reverse transcribed using HiScript III RT SuperMix (Vazyme, Nanjing, China) to synthesize first-strand cDNA.

The full-length coding sequence (CDS) of *RhILL1* was amplified by PCR using 2× Phanta Max Master Mix (Vazyme, Nanjing, China) in a 25 μL reaction volume containing 1 μL cDNA template, 12.5 μL Master Mix, 1 μL each of forward and reverse primers (10 μM), and 9.5 μL nuclease-free water. PCR conditions were as follows: 95 °C for 3 min; 35 cycles of 95 °C for 30 s, 57 °C for 30 s, and 72 °C for 1 min; with a final extension at 72 °C for 5 min.

PCR products were visualized by electrophoresis on a 1.5% agarose gel containing GelRed. Target bands were excised and purified using a Gel Extraction Kit (Omega Bio-Tek, Norcross, GA, USA). Purified fragments were ligated into the pEASY-Blunt Zero Cloning Vector (TransGen Biotech, Beijing, China) and transformed into *Escherichia coli* DH5α competent cells.

Positive colonies were screened by colony PCR using M13 universal primers, and recombinant plasmids were extracted using a Plasmid Mini Kit (Omega Bio-Tek). All positive clones were confirmed by Sanger sequencing (Tsingke Biotechnology, Kunming, China), and the sequences were aligned with the R. chinensis genome to verify the open reading frame (ORF).

### 2.4. Phylogenetic Analysis

The conserved domains of the RhILL1 protein were predicted using the NCBI Conserved Domain Database (https://www.ncbi.nlm.nih.gov/Structure/cdd/wrpsb.cgi) (accessed on 11 October 2023). The RhILL1 protein sequence was aligned using the NCBI BLASTp tool (https://blast.ncbi.nlm.nih.gov/Blast.cgi) (accessed on 21 December 2023) to identify homologs. Protein sequences of ILL1 homologs from other species were retrieved and used to construct a phylogenetic tree with MEGA11 software (Version 11), employing the Neighbor-Joining (NJ) method [28].

### 2.5. Virus-Induced Gene Silencing (VIGS) Assay

The method was adapted from Jing et al. [27]. Based on the verified *RhILL1* sequence, conserved domains were identified, and a 420 bp gene-specific fragment was selected for silencing. The silencing fragment was selected from a region with minimal sequence similarity to *ILL1* family genes in the Rosa genome to ensure target specificity. Primers were designed accordingly, and the fragment was cloned into the pTRV2 vector via homologous recombination using the ClonExpress II One Step Cloning Kit (Vazyme, Nanjing, China), yielding the recombinant plasmid pTRV2-*RhILL1*.

Agrobacterium tumefaciens strain GV3101 was transformed with pTRV1, pTRV2, and pTRV2-*RhILL1* constructs and cultured at 28 °C for 2–3 days until single colonies appeared. The bacteria were grown on LB agar plates supplemented with 50 μg/mL rifampicin and 50 μg/mL kanamycin. Positive clones were verified by colony PCR and cultured overnight in liquid LB medium containing the same antibiotics until OD600 reached 1.5. Bacterial cells were harvested by centrifugation and resuspended in infiltration buffer consisting of 10 mM MgCl_2_, 10 mM MES (pH 5.6), and 100 μM acetosyringone to an OD_600_ of 0.8–1.0.

The pTRV1 strain was mixed 1:1 (*v*/*v*) with either pTRV2 or pTRV2-*RhILL1* suspensions and then incubated at room temperature in the dark for 4–6 h. Vacuum infiltration was performed on detached rose petals under the following conditions: vacuum for 10 min, release for 5 min, repeated for three cycles. Inoculated samples were maintained in the dark at 4 °C for 2–3 days and then transferred to a growth chamber under 16 h light/8 h dark conditions. Petal phenotype was documented every two days, followed by sample collection and phenotype evaluation. Petals were sampled four days post-infiltration for RNA extraction to assess gene silencing efficiency. Petals infiltrated with pTRV2 + pTRV1 served as the negative control. Three biological replicates were used for each treatment group.

### 2.6. Determination of Relative Anthocyanin Content

Anthocyanin extraction and quantification were conducted following the protocol described by Khazaei et al. [29]. Fresh petal tissue (0.1 g) was extracted with 2 mL of 1% (*v*/*v*) HCl/methanol extraction buffer under dark conditions at 4 °C for 24 h. The extract was centrifuged at 12,000 rpm for 1 min at 4 °C. The absorbance of the supernatant was measured at 530 nm and 657 nm using a spectrophotometer (Techcomp UV-2600, Shanghai, China). Relative anthocyanin content was calculated using the formula: ((A530 − A657) × dilution factor/mg FW) × 1000.

### 2.7. Determination of IAA Content

Fresh petal samples were rinsed and ground in liquid nitrogen. A total of 1 mL of pre-chilled extraction buffer (methanol–water–formic acid = 15:4:1, *v*/*v*, containing 10 mg/L BHT) was added to the powdered tissue and incubated at –20 °C overnight. The extract was centrifuged at 15,000× *g* for 30 min at 4 °C. The supernatant was collected, and the pellet was re-extracted and centrifuged again under the same conditions. The two supernatants were combined and evaporated to dryness. The dried residue was dissolved in 300 µL of 1 M methanol.

The IAA concentration was determined using a commercial plant IAA ELISA kit (Zike Biotech, Shenzhen, China), following the manufacturer’s protocol. After several washing and color development steps, absorbance was measured at 450 nm using a microplate reader. The IAA concentration in the samples was calculated based on the standard curve.

### 2.8. Statistical Analysis

All experimental data were based on three independent biological replicates, each with three technical replicates. Statistical analyses were performed using GraphPad Prism 9.5 (GraphPad Software, LLC, San Diego, CA, USA; https://www.graphpad.com) (accessed on 22 April 2024). Differences between the two groups were evaluated using Student’s t-test. Statistical significance was defined as follows: * *p* < 0.05, ** *p* < 0.01, *** *p* < 0.001, and **** *p* < 0.0001.

## 3. Results

### 3.1. Bioinformatic Characterization of RhILL1 in Rose

Using the genome sequence of *Rosa chinensis* ‘Old Blush’ as a reference and cDNA from the cut rose cultivar ‘Pink Floyd’ as a template, the full-length coding sequence of *RhILL1* was successfully cloned and verified by sequencing. The open reading frame (ORF) of *RhILL1* is 1326 base pairs in length, encoding a protein of 441 amino acids. Conserved domain analysis revealed that *RhILL1* contains a Peptidase_M20 domain and an M20_dimer domain, classifying it as a member of the amide hydrolase family. Enzymes in this family participate in IAA metabolism by hydrolyzing amide bonds, thus modulating IAA activity and spatial distribution. Members of the ILR1-like family are typically involved in hormone metabolism and play important roles in regulating plant growth and development [30].

**Figure 1:**
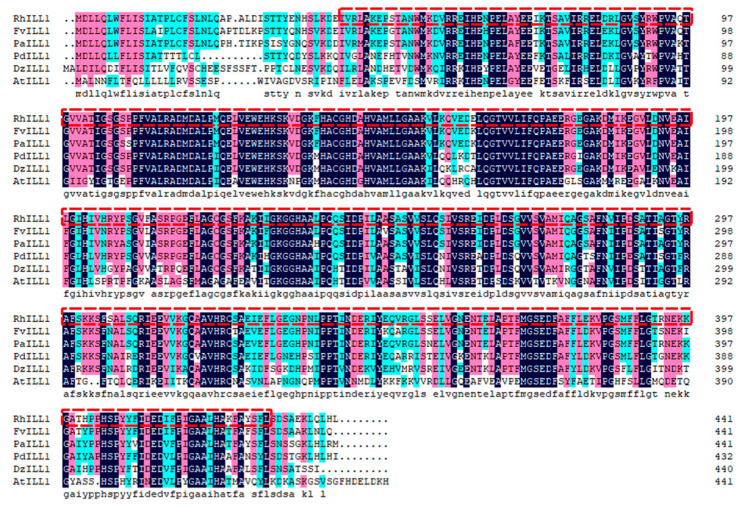
Alignment and analysis of ILL1 protein sequences from various plant species. The box is selected for M20_IAA_Hyd’s conservative domain. Rh: *Rosa hybrida*; Fv: *Fragaria vesca*; Pa: *Potentilla anserina*; Pd: *Prunus dulcis*; Dz: *Durio zibethinus*; At: *Arabidopsis thaliana*.

Multiple sequence alignment of *RhILL1* with five highly similar ILL1 proteins from other species revealed high sequence conservation, suggesting evolutionary conservation of function (Figure 1).

**Figure 2:**
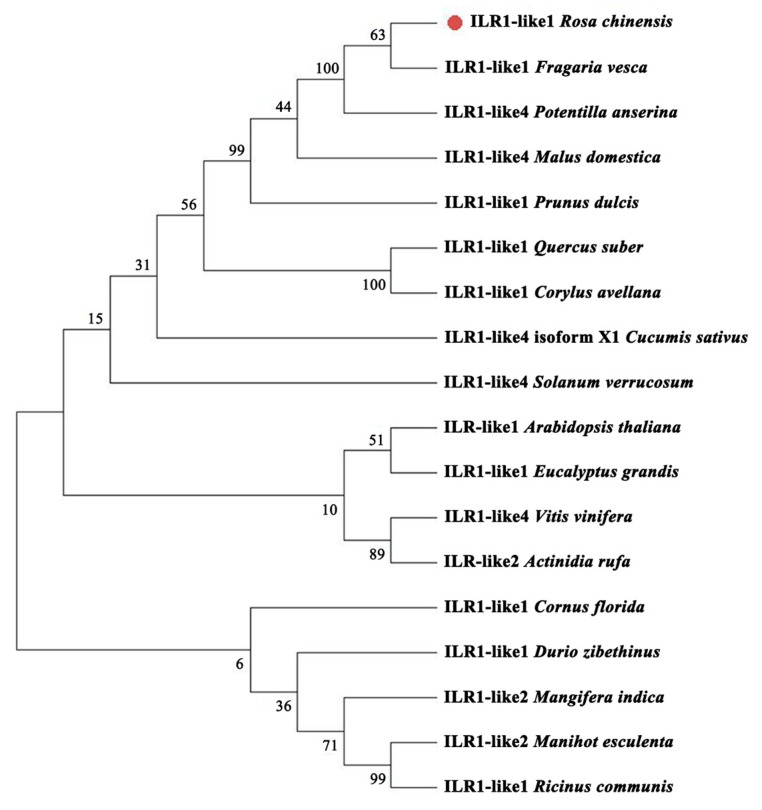
Phylogenetic analysis of RhILL1 with the ILL1 protein of other species.

A phylogenetic tree was constructed using MEGA11 software based on sequence alignment ILL1 homologs from other species (Figure 2). RhILL1 showed the highest sequence similarity to the ILL1 protein from the wild strawberry (*Fragaria vesca*) lineage and was therefore designated as RhILL1 (Figure 2).

### 3.2. Spatiotemporal Expression Pattern of RhILL1

**Figure 3:**
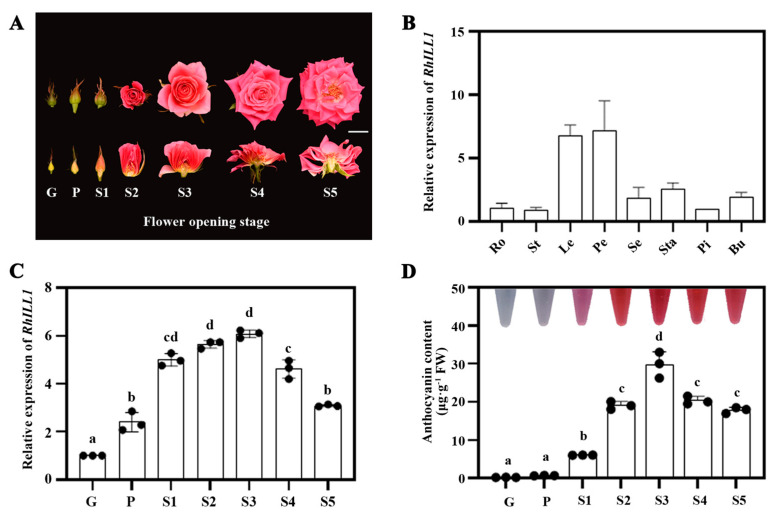
Characteristics analysis of *RhILL1* and anthocyanin content in petals at different developmental stages. (**A**) Phenotypic identification of the ‘Floyd’ rose at various developmental stages. (**B**) Expression levels of *RhILL1* in different tissues of rose, with RhUBI2 used as an internal control. Tissue types include Ro: root, St: stem segment, Le: leaf, Pe: petal, Se: sepal, Sta: stamen, Pi: pistil, and Bu: bud. (**C**) Expression levels of *RhILL1* in rose petals across different floral opening stages. (**D**) Anthocyanin content in petals at various developmental stages. Values with different letters indicate statistically significant differences (one−way ANOVA, *p* < 0.05). Each data point represents at least three biological replicates, presented as means ± standard deviations (SDs).

To investigate the organ-specific expression of *RhILL1* in rose, qRT-PCR analysis was performed using various tissues of ‘Pink Floyd’, including roots, stems, leaves, petals, sepals, stamens, pistils, and buds. As shown in Figure 3B, *RhILL1* was expressed in all the examined organs, with relatively higher transcript levels detected in leaves and petals.

To further explore the spatiotemporal expression profile of *RhILL1* during petal coloration, we examined its expression levels and anthocyanin content across seven developmental stages of ‘Pink Floyd’ petals (Figure 3A). The results revealed that *RhILL1* expression gradually increased in parallel with anthocyanin accumulation, reaching a peak at Stage 3 (S3), and subsequently decreased as anthocyanin levels declined (Figure 3C). These findings suggest a potential correlation between *RhILL1* expression and anthocyanin accumulation in rose petals (Figure 3D).

### 3.3. Silencing of RhILL1 Leads to Petal Color Fading and Alters the Expression of Anthocyanin Biosynthetic Genes

**Figure 4:**
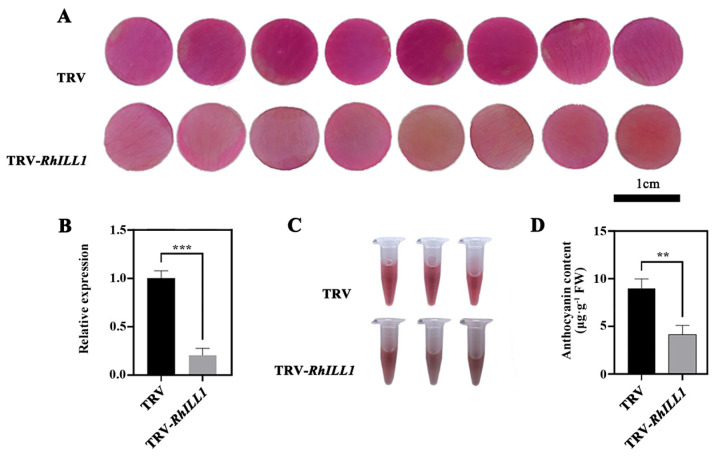
(**A**) Function of *RhILL1* in rose petal color. Phenotypic comparison of petals between TRV and TRV−*RhILL1*; scale bar = 1 cm. (**B**) Detection of silencing efficiency between TRV and TRV−*RhILL1*; scale bar = 2 mm. (**C**) Anthocyanin supernatant of TRV and TRV−*RhILL1*. (**D**) Anthocyanin content in TRV and TRV−*RhILL1*. Significant differences were analyzed using Student’s *t*−test (** *p* < 0.01, *** *p* < 0.001). Data are presented as means ± standard deviations (SDs), with at least three biological replicates for each data point.

To further validate the role of *RhILL1* in petal pigmentation, virus-induced gene silencing (VIGS) was employed to knock down *RhILL1* expression in rose petals. As shown in Figure 4A,B, the *RhILL1*-silenced petals (TRV-*RhILL1*) exhibited visibly lighter pigmentation compared to the control group (TRV). In addition, anthocyanin extracts from the TRV-*RhILL1* petals displayed markedly lighter coloration than those from the TRV controls (Figure 4C).

The quantification of anthocyanin levels further confirmed that the relative anthocyanin content was significantly lower in the *RhILL1*-silenced petals than in the control group (Figure 4D). Collectively, these results indicate that *RhILL1* is positively associated with anthocyanin accumulation and plays a critical role in regulating petal pigmentation in rose.

**Figure 5:**
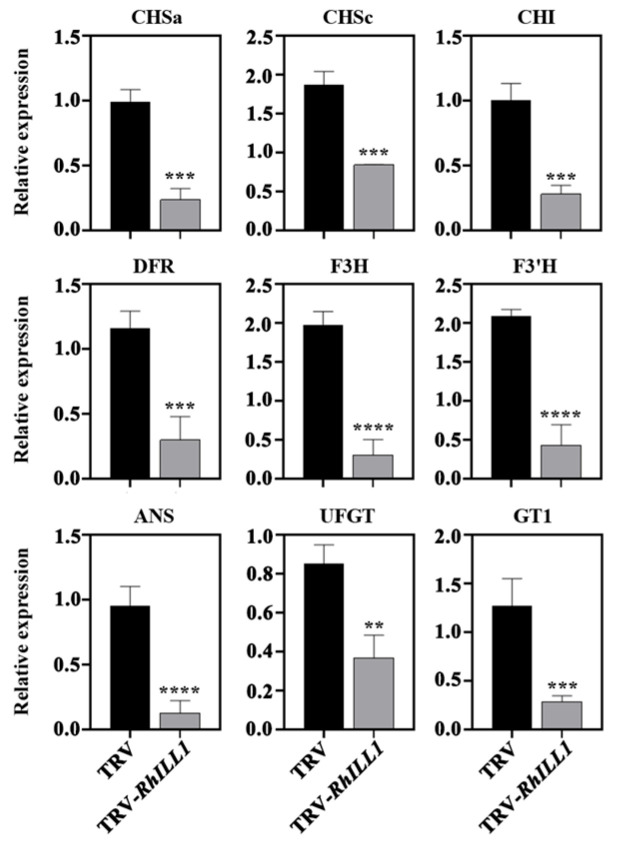
Expression levels of anthocyanin synthesis−related genes after silencing *RhILL1.* Significant differences were analyzed by Student’s *t*−test ((** *p* < 0.01, *** *p* < 0.001, **** *p* < 0.0001), with at least three biological replicates per data, expressed as the mean values ± SDs.

In addition, we quantitatively analyzed the expression levels of key anthocyanin biosynthetic genes in the *RhILL1*-silenced petals and controls. These included early biosynthetic genes (*RhCHS*, *RhCHI*, *RhF3H*) and late biosynthetic genes (*RhDFR*, *RhANS*, *RhUFGT*), as well as *RhF3′H* and *RhGT1*. The results showed that the transcript levels of all these genes were significantly upregulated in the *RhILL1*-silenced petals compared to the TRV controls (Figure 5).

These findings further suggest that *RhILL1* may negatively regulate the expression of structural genes involved in anthocyanin biosynthesis, thereby influencing petal pigmentation in rose.

### 3.4. Silencing of RhILL1 Reduces IAA Levels in Petals and Exogenous Auxin Influences Petal Coloration

**Figure 6:**
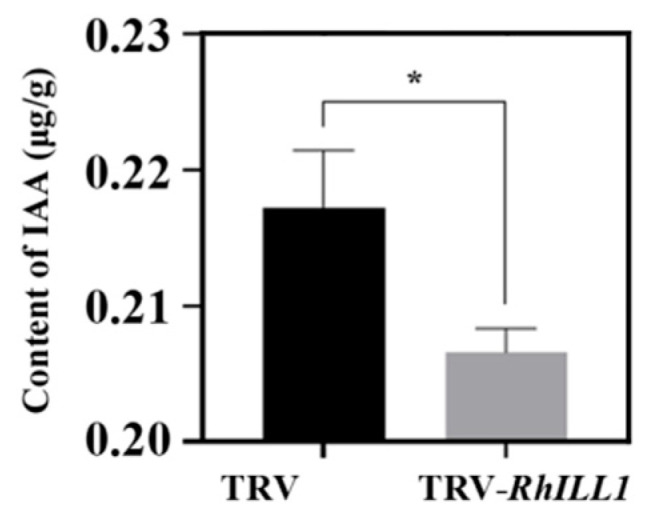
Content of IAA in petals after silencing *RhILL1*. Significant differences were analyzed by Student’s *t*-test (*, *p* < 0.05), with at least three biological replicates per data point, expressed as the mean values ± SDs.

Given that *RhILL1* is a key gene involved in auxin metabolism, we investigated whether its regulatory role in petal pigmentation is associated with IAA. The results showed that the IAA levels were significantly reduced in the *RhILL1*-silenced petals (TRV-*RhILL1*) compared to the control group (TRV) (Figure 6). These findings suggest that *RhILL1* may influence petal pigmentation in rose by modulating endogenous IAA levels.

**Figure 7:**
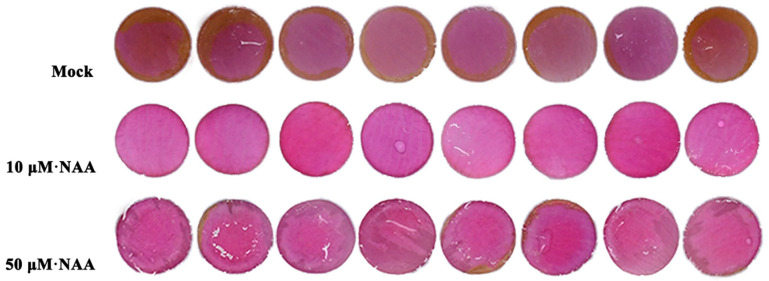
Effect of different concentrations of NAA treatment on petal color.

To further determine the effect of auxin on petal coloration, exogenous treatment with different concentrations of naphthaleneacetic acid (NAA) was applied to the rose petals. Petal phenotypes were assessed 12 days post-treatment. Compared to the mock control, the petals treated with 10 μM NAA displayed deeper coloration and prolonged color retention. Although the petals treated with 50 μM NAA showed slightly lighter coloration than those treated with 10 μM NAA, their pigmentation was still deeper than that of the control (Figure 7). These results indicate that auxin contributes to the maintenance of petal coloration in rose.

## 4. Discussion

Petal coloration in rose involves complex regulatory networks encompassing multiple genes and metabolic pathways, among which auxin and its associated metabolic processes play a critical role. Previous studies have highlighted the importance of phytohormones in flower color regulation [31,32], although the specific involvement of auxin in rose pigmentation remains poorly documented. It has been shown that auxin amide hydrolases, such as ILL1, hydrolyze IAA–amino acid conjugates to release free active IAA, thereby maintaining auxin homeostasis [23]. This hormonal balance is not only essential for normal plant development but also has a direct impact on secondary metabolic processes [33].

In this study, we demonstrated that *RhILL1* expression significantly influences anthocyanin accumulation in rose petals. Silencing *RhILL1* led to reduced expression of key structural genes in the anthocyanin biosynthetic pathway, including *RhCHS*, *RhF3H*, *RhDFR*, and *RhANS*, resulting in visibly lighter petal coloration. These findings indicate that *RhILL1* likely regulates anthocyanin biosynthesis indirectly by modulating endogenous IAA levels. Similar mechanisms have been reported in *A. thaliana* and other model plants, where IAA influences the expression of secondary metabolism-related genes by activating auxin response factors (ARFs) [4,20].

More importantly, ARF transcription factors downstream of IAA signaling can directly bind to the promoters of anthocyanin biosynthetic genes and synergize with MYB regulators to amplify transcriptional responses, thereby enhancing anthocyanin accumulation [4,6]. In tobacco, for example, *NtARF8* has been shown to enhance floral pigmentation [34]. Our previous work has demonstrated that *RhARF8* in rose responds to IAA and, in combination with auxin treatment, suppresses petal fading [35]. In the present study, the regulation of IAA homeostasis by *RhILL1* may activate key transcription factors, such as *RhARF8*, which, in turn, promote the expression of structural genes like *RhCHSa/c*, driving anthocyanin biosynthesis [35].

In addition, *RhILL1* may contribute to prolonged petal color retention through interactions between auxin and ethylene signaling. It has been reported that auxin can delay petal senescence by downregulating the expression of ethylene biosynthesis genes, such as *ACS* and *ACO*, thereby stabilizing anthocyanin levels and maintaining petal vibrancy [12,36,37,38,39]. Based on our findings, we propose that *RhILL1* may influence not only anthocyanin biosynthesis but also petal longevity by antagonizing the ethylene pathway, offering a multifaceted regulatory mechanism for enhancing ornamental quality in rose.

## 5. Conclusions

*RhILL1* functions as a critical regulator of auxin metabolism and signaling. Through the maintenance of IAA homeostasis, modulation of ARF activity, and antagonism of ethylene signaling, *RhILL1* orchestrates anthocyanin biosynthesis and petal color stability. This regulatory mechanism not only advances our understanding of hormone–secondary metabolism interactions in plants but also provides a theoretical framework and genetic resources for molecular breeding aimed at improving floral coloration in rose, with significant implications for ornamental horticulture.

## Data Availability

All data generated or analyzed during this study are included in this published article.

## Supplementary Materials

The following supporting information can be downloaded at: https://www.mdpi.com/article/10.3390/genes16060691/s1, Table S1: Primers used in this study.
