# Supplementary material for: Regulation of Petal Coloration by the Auxin Amide Hydrolase Gene RhILL1 in Rose (Rosa hybrida)
Source: Genes (Basel). 2025 Jun 6;16(6):691. doi: 10.3390/genes16060691 (PMC12192817; doi:10.3390/genes16060691)
Supplement: Supplementary file 1 [file genes-16-00691-s001.zip › genes-3636428-supplementary.pdf]

Supplement Table S1 Primers used in this study

Function	Primer name	Sequence
Virus induced gene silencing	TRV1-F	TTACAGGTTATTTGGGCTAG
	TRV1-R	CCGGGTTCAATTCCTTATC
	TRV2-F	TGGGAGATGATACGCTGTT
	TRV2-R	CCTAAAACTTCAGACACG
	TRV2-*RhILL1*-F	GTGAGTAAGGTTACCGAATTCGAAGTTGCAACTCCATTTGTGAG
	TRV2-*RhILL1*-R	TCTTCGGGACATGGGTACCATAAAAAAACCGGTGTGACACC
qRT-PCR	RhUBI2-F	GCCCTGGTGCGTTCCCAACTG
	RhUBI2-R	CCTGCGTGTCTGTCCGCATTG
	qRT-*RhILL1*-F	CAAGTGCAGTCATTCGTCGC
	qRT-*RhILL1*-R	TCTTGTGCTCCCATTCCACC
	qRT-CHSa-F	CGAGATATCACAATGGTGACCGT
	qRT-CHSa-R	ACATGCGCTGGAATTTCTCCTTGA
	qRT-CHSc-F	CCCAATATCGAAATGGTGACTGTCG
	qRT-CHSc-R	TGGAATTTCTTCTTGAGCTCAACCT
	qRT-CHI-F	CGCCGTTAAGTGGAAGGGT
	qRT-CHI-R	TGGCAGTATCATCGTCACCTG
	qRT-F3H-F：	TTCAAGAACGCCGATCACCA
	qRT-F3H-R：	TGGCTCCTCCAGAATAGGCT
	qRT-F3'H-F：	CTGAAACCTTCAACACACTCGACTC
	qRT-F3'H-R：	ATTTGCCGGAGAAAAGAAGCCG
	qRT-DFR-F：	CGTAGGTTCATGGCTCGTCA
	qRT-DFR-R：	TCCACAGCGTCAAGTGAGTC
	qRT-ANS-F	AGCTCATGGAACGGGTCAAG
	qRT-ANS-R	TTGCCCGGAAGCATTGTTTG
	qRT-UFGT-F	GAGCCACAAAGTTGCTAGTTCTAG
	qRT-UFGT-R	CTTTTTGCCGGAACTCGAGAAG
	qRT-GT1-F	CCTCGACATGTTTTGCGACG
	qRT-GT1-R	ACAGACTGTTGGTTGTGCGA
